# Wearables for health monitoring: body composition estimates of commercial smartwatch and clinical bioelectrical impedance device

**DOI:** 10.3389/fspor.2025.1644082

**Published:** 2025-11-18

**Authors:** Bryson Carrier, Amanda C. Melvin, Jacob R. Outwin, Marni G. Wasserman, Adam P. Audet, Katherine C. Soldes, Kenneth M. Kozloff, Adam S. Lepley

**Affiliations:** 1 University of Michigan, School of Kinesiology, Ann Arbor, MI, United States; 2 The George Washington University, Washington, DC, United States; 3 Indiana University Bloomington, Bloomington, IN, United States; 4 Washington University in St. Louis, St. Louis, MO, United States; 5 Department of Orthopaedic Surgery, Michigan Medicine, University of Michigan, Ann Arbor, MI, United States

**Keywords:** wearable technology, activity monitor, biometric technology, body fat percentage, skeletal muscle mass, dual-energy x-ray absorptiometry

## Abstract

**Introduction:**

Body composition is a critical health measure. Accurate monitoring of body composition, such as body fat percentage (BF%) and skeletal muscle mass percentage (SM%), enables individuals to make informed decisions regarding nutrition, exercise, health status and management. Recent advancements have integrated bioelectrical impedance analysis (BIA) into wearable technology, presenting accessible options for tracking body composition measures. However, the validity of wearable BIA devices in comparison to criterion methods remains underexplored. Therefore, this study aimed to assess the validity of a wrist-worn consumer device and a clinical BIA device against the criterion measure of dual-energy x-ray absorptiometry (DXA).

**Methods:**

This study included 108 physically active participants (56 females, 52 males). Participants underwent assessments using DXA, a wearable smartwatch BIA device (wearable-BIA; Samsung Galaxy Watch5), and a clinical standing hand-to-foot BIA analyzer (clinical-BIA; InBody 770). Measures of interest included BF% and SM%. Data were analyzed for accuracy using tests of error [mean absolute error [MAE], mean absolute percentage error [MAPE]], linearity (Pearson's *r*, Deming regression), agreement (Lin's CCC), and equivalence, complemented by Bland-Altman plots to visually represent bias.

**Results:**

When assessing BF%, both the wearable-BIA (*r* = 0.93; CCC = 0.91) and clinical-BIA (*r* = 0.96; CCC = 0.86), demonstrated very strong correlations and agreement compared to DXA, with MAPEs of 14.3% and 21.1%, respectively. Female participants using the wearable-BIA device showed the greatest accuracy for BF% (CCC = 0.91, MAPE = 9.19%, equivalence supported). Bland-Altman analysis revealed proportional bias, particularly in individuals with higher BF%. Although correlations were considered strong for SM%, agreement was classified as weak (wearable-BIA: *r* = 0.92, CCC = 0.45; MAPE = 20.3%; clinical-BIA, *r* = 0.89; CCC = 0.25; MAPE = 36.1%).

**Discussion:**

Both the wearable- and clinical-BIA device revealed mixed validity, demonstrating strong correlations for both BF% and SM%, and high levels of agreement and low error for BF%. Additionally, the wearable-BIA demonstrated acceptable accuracy for estimating BF% in females. However, wider limits of agreement and variability suggest limitations in validity, particularly for skeletal muscle mass and in individuals with higher body fat percentages. These findings support the practical use of wearable devices for general body composition monitoring when laboratory-based methods are unavailable, though caution is warranted. Continued development and validation efforts are recommended to enhance accuracy and consistency across diverse populations and measures.

## 1 Introduction

Body composition outcomes are key health measures in understanding an individual's overall health and fitness status, providing critical insights into the balance between fat and lean tissue, which can influence metabolic health and physical performance (1). Analysis of body composition, including body fat percentage (BF%) and skeletal muscle percentage (SM%) offers a more nuanced view of health by differentiating between fat and lean mass, as compared to more simple but easily accessible measures such as body mass or body mass index (BMI) (2–5). This distinction can be important, as two individuals with the same BMI can have vastly different health profiles depending on their fat distribution and muscle mass. Tracking BF% and SM% over time is relevant not only for identifying potential risk profiles and metabolic health in the general population, it also can be used to monitor training outcomes and progress toward goals in those who are physically active. While the role of body composition in sport performance is variable, and unique to the sport being performed, the ability to accurately track compositional changes over time is of great interest to athletes, coaches, physically active individuals, and those monitoring weight changes (6–10). Regular monitoring of body composition can help individuals make informed decisions about adjusting their diet, exercise, and lifestyle to achieve and maintain their health and fitness goals (1).

There are various methods currently available to assess body composition. These devices range from simple and cost-effective equations based on anthropometric measurements, which are prone to high error, to criterion measurements, such as the dual-energy x-ray absorptiometry (DXA) known for its accuracy and reliability (11). Other high-quality methods, such as hydrostatic weighing and air displacement plethysmography, require specialized equipment and facilities, making them time consuming, expensive, and relatively inaccessible (12). More accessible options, like skinfold measurements and bioelectrical impedance analysis (BIA) are common due to their ease of use and affordability (13). Skinfold measurements are convenient and low cost; however, they require a high degree of training to achieve proper technique and have greater error than other methods (14). BIA, by contrast, has become an appealing method for assessing body composition because it is non-invasive, quick, cost-effective, and relatively accurate, making it accessible for both in-clinic and at-home use while providing immediate feedback on body composition measures (15).

BIA provides estimates of body composition by measuring the resistance of body tissues to a low-level electrical current (16). Current BIA devices are manufactured using various configurations, including at-home BIA scales, clinical hand-to-foot analyzers, and advanced octopolar systems. Advances in technology have made many biomonitoring sensors more practical for everyday health monitoring. Recently, companies have begun integrating BIA technologies into commercially available wearable devices (smartwatches), offering the potential of an accurate, convenient, and accessible solution for monitoring body composition and enhancing public engagement in health monitoring. These devices can deliver frequent estimations of body composition through non-invasive means, providing users with actionable information about their health and fitness status. However, many of these devices have yet to undergo comprehensive independent validation to assess their accuracy compared to traditional laboratory methods (15). Additionally, as wearable technologies continue to evolve, updates to hardware design, sensor configuration, and proprietary algorithms are common across device generations. Accordingly, independent validation of new models is critical to ensure that previously established levels of accuracy remain valid and that findings from earlier versions can be appropriately generalized to newer devices. Independent validation is essential for consumers to rely on these devices for individual health monitoring. Thus, the purpose of this study is to assess the accuracy of a wrist-worn wearable device utilizing BIA technology to estimate body fat percentage and skeletal muscle percentage compared to DXA and established clinical BIA methods.

## 2 Materials and methods

A total of 108 participants were enrolled in the study, including 56 females and 52 males (self-reported gender). Participants were included if they were between the ages of 18–80 and participated in moderate to vigorous physical activity at least three days per week. Participants were excluded if they had a contraindication to intense exercise (e.g., cardiovascular disease, significant musculoskeletal or neurological impairments, etc.) or were pregnant. All participants provided written informed consent prior to testing, and all procedures were approved by the University's Institutional Review Board (IRB#: HUM00220366). Participants underwent body composition assessments during a single visit, where they were required to wear lightweight athletic clothing. Prior to their visit, participants were instructed to consume water like they normally would and to refrain from food, caffeine, or other drink for 3 h prior to their appointment as per device instructions. Participants were also instructed to avoid alcohol, smoking and heavy exercise for 24 h prior to their visit.

**Figure 1:**
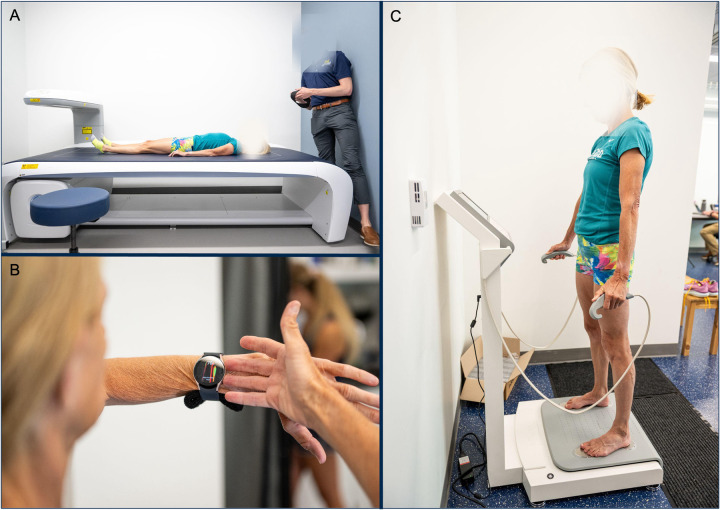
image of methodology to collect body composition from **(A)**. DXA, **(B)**. wearable smartwatch BIA (wearable-BIA) and **(C)**. clinical standing hand-to-foot BIA analyzer (clinical-BIA).

During the visit, body composition was measured using three different methods for comparison: DXA, wearable smartwatch BIA device (wearable-BIA), and a clinical standing hand-to-foot BIA analyzer (clinical-BIA). Criterion measurements were obtained using a total body DXA scan (Lunar iDXA, General Electric, Boston, MA, USA; enCORE v18 software). The wearable-BIA (Samsung Galaxy Watch5, Samsung Electronics Co. Ltd., Seoul, South Korea) employs BIA through two metal knobs on the watch. Per device instructions, after demographic information was input, the participants were instructed to place their middle and ring fingers from one hand on these knobs for 30 s to 1 min to obtain body fat percentage readings. At the time of our study, no other commercially available smartwatches included wearable BIA assessment, thus we chose this device to be compared to a criterion standard and a clinical device using BIA to assess body composition. The clinical-BIA (InBody 770, InBody Co Ltd., Seoul, South Korea) was used as an additional clinical comparison. For this assessment, the participants were positioned on a standing hand-to-foot BIA analyzer following device instructions (Figure 1). Importantly, at the time of our study, no other commercially available smartwatches included wearable BIA assessment. This underscores the novelty of the technology and highlights why it was essential to rigorously evaluate its validity at an early stage. Establishing this evidence base is critical not only for the sponsor, but also for consumers, clinicians, and researchers who are increasingly using these devices to monitor and track body composition. By comparing the smartwatch to both DXA, the criterion standard, and a widely used clinical device (InBody), our study provides a foundation for interpreting results from a novel technology that is rapidly entering both consumer and research settings.

Fat and skeletal muscle mass (kg), and BF% and SM% values were directly reported by each device for further analysis.

### 2.1 Data analysis

Participant data was collected and managed via spreadsheet software and a web-based data management system (REDCap, Vanderbilt University, Nashville, TN) hosted at University of Michigan (17). Measures for body composition were obtained as cross-sectional data provided by the test devices after each trial. All statistics were run in jamovi [The jamovi project (2024). Jamovi (Computer Software). Version 2.6.19, retrieved from https://www.jamovi.org]. Equivalence plots were plotted using an R Shiny app (R Statistical Software) (18).

Mean differences between the three devices in reported fat and skeletal muscle mass (kg) values were compared using a one-way ANOVA with Bonferroni *post hoc* comparisons. Accuracy for BF% and SM% was determined via tests of error, linearity, agreement, and equivalence, with visual representation of bias shown with Bland-Altman plots (19–22). Statistics were performed for the overall sample, as well as for gender and weight stratifications. The wearable-BIA and clinical-BIA devices were used as the test measurements for all statistical tests, and DXA as the criterion measurement. Mean absolute error (MAE) and mean absolute percentage error (MAPE) were calculated for error analysis. Agreement and linearity were established via Lin's Concordance Correlation Coefficient (CCC), Pearson's Product Moment Correlation (*r*), and Deming Regression. Correlation coefficients were interpreted as follows: 0 to <0.2, very weak; ≥0.2 to <0.4, weak; ≥0.4 to <0.6, moderate; ≥0.6 to <0.8, strong; and ≥0.8–1.0, very strong (23). Equivalence testing was conducted using 90% confidence intervals, consistent with the standard approach at *α* = 0.05. In this framework, a 90% CI that lies entirely within the predefined equivalence bounds indicates statistical equivalence at the 5% level and is broadly accepted in equivalence testing literature (18). In addition to descriptive statistics, combined validity criteria were set at MAPE < 10%, CCC > 0.7, and equivalence supported at 10% (±5%) of the criterion mean for equivalence window, based on the 90% CI (20, 24). Binary results for the equivalence testing can be found in the validity statistics.

## 3 Results

A total of 108 participants completed the body composition assessments; demographic summary statistics can be found in Table 1. ANOVA revealed significant differences in both fat mass [DXA = 18.1 ± 9.6 kg; wearable-BIA = 19.0 ± 8.9 kg; clinical-BIA = 15.4 ± 9.7 kg; *F*(2, 323) = 4.2, *p* = 0.01] and skeletal muscle mass [DXA = 23.1 ± 5.4 kg; wearable-BIA = 27.4 ± 5.7 kg; clinical-BIA = 31.2 ± 6.6 kg; *F*(2, 323) = 49.9, *p* < 0.001] across the three devices. Bonferroni *post hoc* analyses indicated that all devices differed significantly from each other in skeletal muscle mass estimates (*p* < 0.05), whereas for fat mass, a significant difference was observed only between the wearable-BIA and clinical-BIA devices (*p* = 0.01). Neither the wearable-BIA (*p* = 1.0) or clinical-BIA (*p* = 0.10) device was significantly different from DXA.

**Table 1:** Participant demographics reported as ± standard deviation. Fat mass, body fat percentage (BF%), skeletal muscle mass, and skeletal muscle percentage (SM%) measures are reported from DXA results.

Group	Age (yrs)	Height	Weight (kg)	BMI (kg/m^2^)	Fat mass (kg)	BF%	Skeletal muscle mass (kg)	SM%
Overall (*n* = 108)	39.3 ± 13.7	171.0 ± 9.0	70.6 ± 14.8	24.0 ± 4.3	18.1 ± 9.6	25.4 ± 9.3	23.1 ± 5.4	32.6 ± 9.7
Male (*n* = 52)	41.1 ± 14.5	177.4 ± 7.1	75.8 ± 13.2	24.0 ± 3.8	15.6 ± 8.3	20.3 ± 7.8	27.9 ± 4.4	35.7 ± 3.2
Female (*n* = 56)	37.7 ± 12.9	165.0 ± 6.0	65.7 ± 14.6	24.0 ± 4.7	20.4 ± 10.2	30.1 ± 8.2	19.5 ± 3.2	29.7 ± 3.5

**Figure 2:**
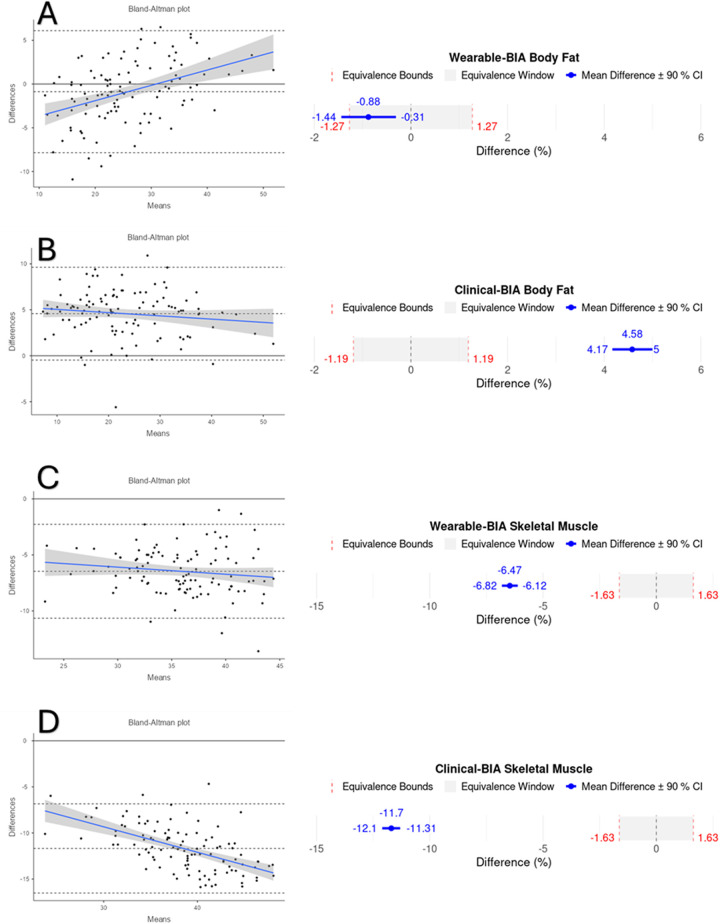
Bland-Altman and equivalency plots for combined BF% and SM% data for wearable-BIA and clincal-BIA devices compared to criterion measurement (DXA). Wearable-BIA BF% results found in panel **(A)**, Clinical-BIA BF% results found in panel **(B)**. Wearable-BIA SM% results found in panel **(C)**, Clinical-BIA SM% results found in panel **(D)**. Blue lines on Bland-Altman plots represent the proportional bias line with shadings representing 95% confidence intervals of proportional bias line. *X*-axis is the mean of the two measurements with the *Y*-axis the difference between the two measurements. The mean bias line and upper and lower limits of agreement are shown in dashed lines (mean bias being the middle-dashed line). The solid line represents the hypothetical mean bias of 0. Equivalence window for equivalence plots determined based on 10% of criterion mean (±5%).

**Figure 3:**
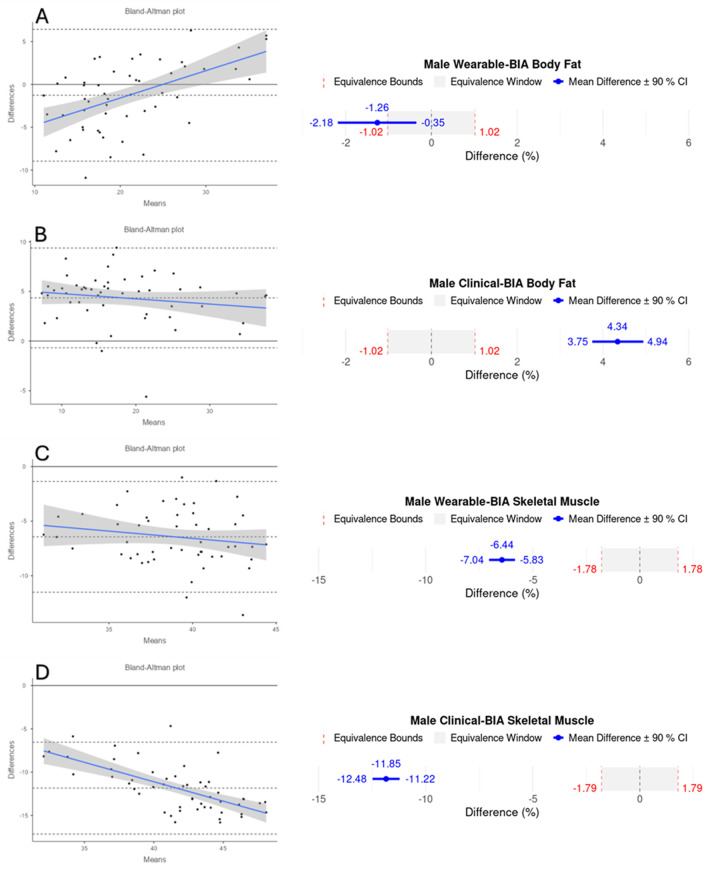
Bland-Altman and equivalency plots for male participant BF% and SM% data for wearable-BIA and clinical-BIA devices compared to criterion measurement (DXA). Wearable-BIA BF% results found in panel **(A)**, Clinical-BIA BF% results found in panel **(B)**. Wearable-BIA SM% results found in panel **(C)**, Clinical-BIA SM% results found in panel **(D)**. Blue lines on Bland-Altman plots represent the proportional bias line with shadings representing 95% confidence intervals of proportional bias line. *X*-axis is the mean of the two measurements with the *Y*-axis the difference between the two measurements. The mean bias line and upper and lower limits of agreement are shown in dashed lines (mean bias being the middle-dashed line). The solid line represents the hypothetical mean bias of 0. Equivalence window for equivalence plots determined based on 10% of criterion mean (±5%).

**Figure 4:**
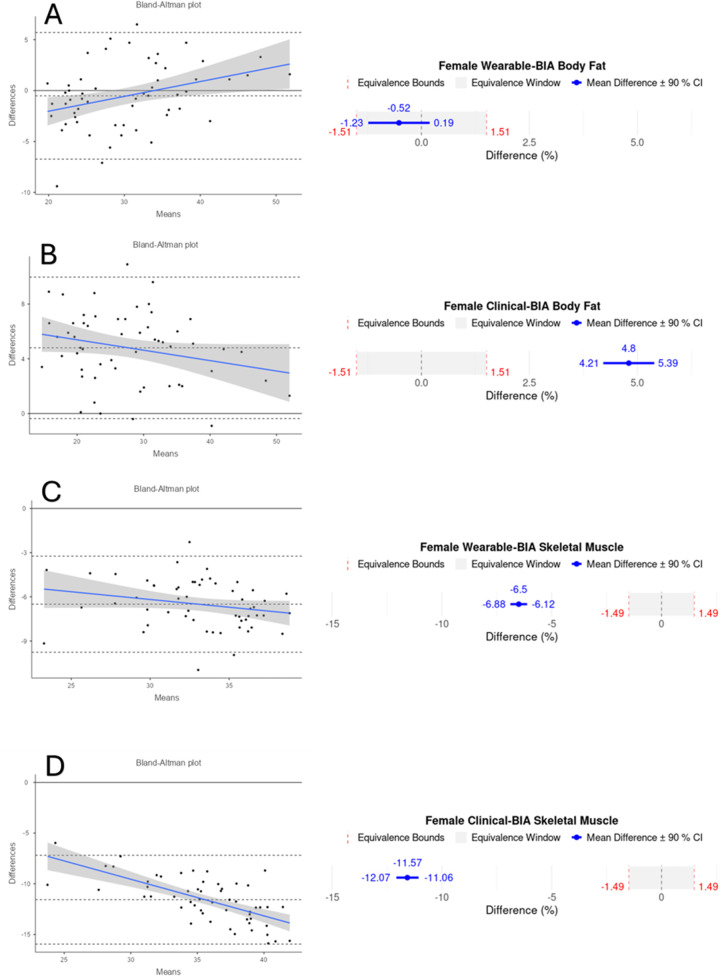
Bland-Altman and equivalency plots for female participant BF% and SM% data for wearable-BIA and clinical-BIA devices compared to criterion measurement (DXA). Wearable-BIA BF% results found in panel **(A)**, Clinical-BIA BF% results found in panel **(B)**. Wearable-BIA SM% results found in panel **(C)**, Clinical-BIA SM% results found in panel **(D)**. Blue lines on Bland-Altman plots represent the proportional bias line with shadings representing 95% confidence intervals of proportional bias line. *X*-axis is the mean of the two measurements with the *Y*-axis the difference between the two measurements. The mean bias line and upper and lower limits of agreement are shown in dashed lines (mean bias being the middle-dashed line). The solid line represents the hypothetical mean bias of 0. Equivalence window for equivalence plots determined based on 10% of criterion mean (±5%). Equivalency test supported for Females wearable-BIA BF% condition only.

Validity statistics for BF% and SM% can be found in Table 2 for combined data and Table 3 for gender stratified data. Accompanying plots are depicted in Figures 2–4. When assessing BF%, results showed very strong correlations for both the wearable-BIA and clinical-BIA compared to DXA. Validity criteria for BF% accuracy were met for agreement for both devices (wearable-BIA CCC = 0.91, clinical-BIA CCC = 0.86) but were not met for error (wearable-BIA MAPE: 14.30%, clinical-BIA MAPE: 21.19%) or equivalence testing. The wearable-BIA met validity thresholds for female BF% estimation (CCC = 0.91, MAPE = 9.19%, Equivalence Test = Supported). When assessing SM%, results also showed very strong correlations for both the wearable-BIA and clinical-BIA compared to DXA. Validity criteria for SM% accuracy were not met for agreement (wearable-BIA CCC = 0.45, clinical-BIA CCC = 0.25), error (wearable-BIA MAPE: 20.3%, clinical-BIA MAPE: 36.1%), or equivalence testing for overall or gender stratified data. Overall, Bland-Altman analyses revealed wide limits of agreement across both BF% and SM% outcomes, suggesting a wide range of variability. Additionally, the plots demonstrated proportional bias, with differences increasing with those who demonstrate higher BF% estimates. Additional validity statistics stratified by weight can be found in the supplementary files (Supplementary Table S1).

**Table 2:** Validity statistics for overall data, wearable-BIA (samsung galaxy Watch5) and clinical-BIA (inBody 770) compared to dual-energy x-Ray absorptiometry (DXA) for estimating body fat percentages (BF%), and skeletal muscle percentages (SM%).

Variable	Device	*n*	Mean ± SD	MAE	MAPE	*r*	CCC	Slope	Intercept	Eq.	Bias (95% CI)	LOA
Body fat (%)	Wearable-BIA	108	26.30 ± 7.93	2.87	14.36	0.93	0.91	0.83	5.11	No	−0.88 (−1.55, −0.20)	−7.85, 6.1
	Clinical-BIA	108	20.84 ± 9.73	4.73	21.33	0.96	0.86	1.04	−5.55	No	4.58 (4.09, 5.07)	−0.47, 9.63
Skeletal muscle (%)	Wearable-BIA	106	37.11 ± 4.78	6.47	20.33	0.92	0.45	1.07	4.23	No	−6.46 (−6.87, −6.05)	−10.65, −2.27
	Clinical-BIA	107	44.38 ± 5.86	11.7	36.14	0.89	0.25	1.33	0.83	No	−11.68 (−12.15, −11.21)	−16.52, −6.84

MAE, mean absolute error; MAPE, mean absolute percentage error; CCC, Lin's concordance correlation coefficient; *r*, Pearson's product MOMENT correlation; Eq., equivalence test (supported yes/no); CI, confidence interval; LOA, limits of agreement (reported as lower, upper).

**Table 3:** validity statistics for gender stratified data, wearable-BIA (samsung galaxy Watch5) and clinical-BIA (inBody 770) compared to dual-energy x-Ray absorptiometry (DXA) for estimating body fat percentages (BF%), and skeletal muscle percentages (SM%).

Gender	Outcome	Device	n	Mean ± SD	MAE	MAPE	*r*	CCC	Slope	Intercept	Eq.	Bias (95% CI)	LOA
Male	Body fat (%)	Wearable-BIA	52	21.64 ± 5.79	3.26	19.92	0.88	0.82	0.71	7.18	No	−1.26 (−2.36, −0.17)	−8.96, 6.43
		Clinical-BIA	52	16.03 ± 8.24	4.61	25.66	0.95	0.83	1.06	−5.47	No	4.34 (3.63, 5.06)	−0.68, 9.37
	Skeletal muscle (%)	Wearable-BIA	51	42.14 ± 3.69	6.44	18.33	0.73	0.27	1.17	0.33	No	−6.44 (−7.17, −5.71)	−11.51, −1.37
		Clinical-BIA	52	47.56 ± 4.99	11.85	33.15	0.87	0.16	1.63	−10.56	No	−11.85 (−12.60, −11.09)	−17.15, −6.54
Female	Body fat (%)	Wearable-BIA	56	30.63 ± 7.18	2.51	9.19	0.92	0.91	0.86	4.76	Yes	−0.52 (−1.37, 0.33)	−6.74, 5.71
		Clinical-BIA	56	25.32 ± 8.90	4.85	17.32	0.96	0.82	1.08	−7.24	No	4.80 (4.08, 5.51)	−0.37, 9.97
	Skeletal muscle (%)	Wearable-BIA	55	36.29 ± 3.89	6.5	22.19	0.9	0.35	1.12	3.01	No	−6.50 (−6.95, −6.05)	−9.76, −3.24
		Clinical-BIA	55	41.36 ± 5.00	11.57	38.95	0.92	0.19	1.46	−2.21	No	−11.57 (−12.17, −10.96)	−15.95, −7.19

MAE, mean absolute error; MAPE, mean absolute percentage error; CCC, Lin's concordance correlation coefficient; *r*, Pearson's product moment correlation; Eq., equivalence test (supported yes/no); CI, confidence interval; LOA, limits of agreement (reported as lower, upper).

## 4 Discussion

The current study aimed to evaluate the accuracy of a smartwatch utilizing BIA technology for estimating body composition. The ability to accurately assess body composition using a smartwatch offers significant advantages, including on-demand monitoring and improved accessibility without the need for frequent laboratory visits or DXA-associated ionizing radiation exposure. This capability can provide users with timely and actionable insights into their health and fitness, supporting informed decisions about diet, training, and overall health management. This study discovered significant mean differences in skeletal muscle mass (kg) observed across all devices. While both the wearable-BIA and clinical-BIA devices produced fat mass (kg) estimates similar to DXA, they differed significantly from each other. These findings support the broader validity results, discussed below, highlighting that while group-level fat mass estimates were generally comparable between DXA and wearable-BIA, significant discrepancies remain between BIA devices, particularly for skeletal muscle mass. The observed differences across all three devices for skeletal muscle mass underscore the limited agreement and high variability seen in other validity metrics, reinforcing the caution needed when interpreting absolute values from BIA devices.

Both the wearable- and clinical-BIA device revealed mixed validity, demonstrating strong correlations for both BF% and SM%, and high levels of agreement and low error for BF%. Notably, BF% estimation for female participants met all accuracy thresholds, including low MAPE, high correlation, and equivalence with DXA values. This finding suggests that female users may have greater confidence in the wearable-BIA device's measurements, highlighting its potential as a practical and reliable tool for body composition monitoring in this population. It is important to note that the thresholds utilized for the current investigation of MAPE < 10%, CCC > 0.7, and equivalence supported at a window of 10% (±5%) based on 90% CI's, are relatively liberal, and chosen as we believe it is sufficiently stringent for testing consumer-grade devices for recreational use. As acceptable thresholds have not been widely established (24), those looking to utilize these devices for other purposes (athletics, research, clinical, etc.) may choose to apply more stringent validity thresholds than what we have chosen.

BIA devices estimate body composition by measuring the impedance/conductance of low-level electrical currents through body tissues, relying on proprietary algorithms to generate these estimates. While the literature has consistently shown that BIA devices exhibit errors in measuring body composition when compared to criterion DXA assessments, advancements in technology have improved their accuracy and accessibility (15, 25). In this study, both the wearable- and clinical-BIA devices demonstrated errors in their estimations compared to DXA. While the MAEs for the wearable-BIA (BF% = 2.8%; SM% = 6.4%) and clinical-BIA (BF% = 4.3%; SM% = 11.7%) devices were relatively small, indicating the average absolute difference from DXA-measured values, the corresponding MAPEs are substantially larger (wearable-BIA: BF% = 14.3%; SM% = 20.3%; clinical-BIA: BF% = 21.3%; SM% = 36.1%). This discrepancy highlights a key limitation of MAPE when used with percentage-based variables like BF% and SM%, where small absolute differences can yield large relative percentage errors due to the proportional scaling. Therefore, it is important to interpret MAPE values in conjunction with MAE to better understand the practical magnitude of error. These MAE values align with prior reports on the accuracy of wearable and clinical BIA technologies. (26, 27).

As mentioned above, many BIA devices utilize proprietary algorithms that indirectly estimate body composition from electrical conductivity of the body, among other measures. It has been suggested that BIA-derived outputs may not provide valid results if the underlying prediction equations are not appropriately selected or calibrated based on factors such as sex, age, race, and body size (28). However, the specific algorithms used by most commercial systems, including wearable BIA devices, are not publicly disclosed. This lack of transparency prevents the scientific community from fully evaluating how input signals are processed or weighted, identifying potential sources of systematic error, or proposing refinements that might improve generalizability across populations. The inability to access or replicate these proprietary algorithms limits scientific reproducibility and constrains the interpretability of validation findings. While studies such as ours can rigorously assess applied validity, that is, the degree to which device outputs correspond with criterion measures under controlled conditions, they cannot isolate which aspects of the algorithm or hardware contribute to observed discrepancies. Consequently, results should be viewed as an evaluation of overall device performance rather than a mechanistic validation of the underlying model. Despite this limitation, independent validation remains scientifically valuable because it provides transparent, empirical evidence regarding real-world measurement accuracy, helps identify consistent sources of bias across device generations, and informs researchers and clinicians on the practical reliability of wearable technology when algorithmic details remain opaque. And while a complete analysis of BIA estimation equations is outside the scope of the current study, we believe it is important to note it as a limitation inherent with testing the validity of consumer-grade devices where proprietary algorithms are not disclosed and changes and updates to the models are inevitable. With the above said, and based on the output of the included devices, we speculate that the models are a 3-compartment model, similar to the criterion DXA assessment. As stated earlier, the only group that the device provided sufficiently accurate results were female BF% estimation, where female participants demonstrated high correlation and low error, and equivalency testing deemed the wearable-BIA comparable to DXA BF% values. Thus, based on our results, females can expect acceptably accurate results with less than 10% error (in terms of MAPE) and 2.51% (in terms of MAE) in their BF% estimate when using this device. Although the reason for the higher accuracy of female BF% measurements compared to males remains unclear, previous research has also highlighted differences in BIA assessment accuracy between genders. This discrepancy may be attributed to variations in fat and fat-free mass distribution between genders, as well as potential algorithmic adjustments for gender that are not fully disclosed across different devices (29). Further research is needed to better understand these influencing factors and enhance the accuracy of BIA across different populations.

One of the key strengths of our study is the inclusion of a large and diverse participant pool. Specifically, participants varied in age (18–77 years), BMI (17.70–41.38 BMI), and DXA BF% values (8.60%–52.60%). This broad representation enhances the generalizability and applicability of our findings. There were, however, notable biases detected, particularly for individuals with higher body fat percentages using the wearable-BIA. The Bland-Altman plots display proportional bias where the difference between devices increases with higher BF% estimates. This indicates increasing disagreement between devices at higher measurement ranges, limiting their interchangeability for individuals with higher BF%. These biases also suggest that while the wearable devices can provide general trends in body composition, they may lack the precision required for clinical assessments or detailed monitoring, especially in cases where small changes are significant for training adjustments or health interventions. It should be noted that this study only investigated these devices during a one-time, cross-sectional assessment. Future research should evaluate the effectiveness of these devices to detect changes over time in the same individuals, which would provide users additional confidence in the measurements and the devices ability to detect longitudinal changes in body composition measures.

It should be noted that the wearable device assessed in this study represents a newer generation of a model that has previously incorporated wearable-BIA technology and has been assessed for accuracy (Samsung Galaxy Watch 4 vs. Watch 5). Although manufacturers rarely disclose the specific nature of hardware or algorithmic updates between device iterations, available marketing material for the Galaxy Watch 5 indicates refinements to the bioimpedance sensors and wrist electrode interface, suggesting that independent validation of each new generation remains warranted. In the present study, the Galaxy Watch 5 demonstrated similar levels of correlation, error and agreement with criterion measures compared to those previously reported for the Galaxy Watch 4 (26), indicating comparable performance across models. Importantly, our analysis is the first to examine gender-specific and weight-stratified accuracy for any wearable-BIA device, providing new insight into potential demographic influences on measurement validity. Given that even minor modifications in sensor design or signal-processing algorithms can meaningfully alter impedance-derived estimates, future generations of this and similar consumer wearables should be independently re-evaluated. Routine validation of each hardware and software generation aligns with best practice recommendations (30, 31) for consumer device evaluation and supports transparency and reproducibility as these technologies continue to evolve.

While discrepancies between wearable-BIA estimates and DXA measurements highlight current limitations in wearable-derived body composition estimates, it is noteworthy that the wearable-BIA performed comparably to, and in some cases better than, the clinical-BIA device as indicated by lower MAE and MAPE values, and higher correlation and agreement statistics. Previous research has shown that wearable-derived BIA measurements exhibit similar levels of agreement with other BIA methods, such as octopolar BIA (26). These findings collectively suggest that the inherent limitations of BIA as a technique may be consistent across specific types of BIA used. This is particularly relevant as standard clinical and commercial body composition analyzers are costly and often inaccessible. Consumers can therefore feel confident in achieving similar levels of accuracy with wearable BIA devices. Smartwatch-derived body composition estimates offer a practical and accessible alternative, or at the very least a complementary tool, particularly when higher-accuracy methods are unavailable or infeasible, or for self-monitoring between clinical visits.

While we were able to include a relatively large and diverse sample, a limitation of the current study is that we only included healthy individuals who were considered physically active. Our data should be interpreted as such, and further research is needed to confirm if accuracy values detected here can also be applied to other populations, such as sedentary individuals or those with cardiovascular or metabolic medical conditions. Additionally, our results can only be applied to the wearable device used in this study. As mentioned above, technology and proprietary algorithms adapt and evolve over time, therefore regular independent evaluation is important for the ever-evolving hardware and sensor changes in wearable technology. Lastly, although all three devices were used during a single, same-session visit, ensuring concurrent measurements within each participant, we did not standardize the time of day for the visit across participants. As a result, we cannot determine the potential influence of testing time or diurnal variation on device agreement.

In conclusion, the wearable-BIA device assessed in this study performed comparably to the clinical-BIA device and demonstrated acceptable accuracy for estimating BF% in females compared to the criterion DXA values. However, the broader findings, especially for skeletal muscle mass and in those with greater BF%, highlight variability and limitations in validity that restrict the use of these devices for precise, individual-level assessments. While not a valid replacement for laboratory-based methods, wearable-BIA may offer a practical, accessible alternative for general monitoring in recreational settings. Future research should further examine the device's ability to track changes over time and validate its accuracy in more diverse populations to strengthen confidence in its use for health and fitness monitoring.

## Data Availability

The raw data supporting the conclusions of this article will be made available by the authors, without undue reservation.
