# Supplementary material for: Wearables for health monitoring: body composition estimates of commercial smartwatch and clinical bioelectrical impedance device
Source: Front Sports Act Living. 2025 Nov 18;7:1644082. doi: 10.3389/fspor.2025.1644082 (PMC12669227; doi:10.3389/fspor.2025.1644082)
Supplement: Supplementary file 1 [file Table1.docx]

Supplementary Table 1. Validity statistics for weight stratified data (<60kg, 60-70kg, 70-80kg, 80-90kg, >90kg), wearable-BIA (Samsung Galaxy Watch5) and clinical-BIA (InBody 770) compared to dual-energy X-Ray absorptiometry (DXA) for estimating body fat percentages (BF%) and skeletal muscle percentages (SM%). MAE = Mean Absolute Error, MAPE = Mean Absolute Percentage Error, CCC = Lin’s Concordance Correlation Coefficient, r = Pearson’s Product Moment Correlation, CI = Confidence Interval, LOA = Limits of Agreement (reported as lower, upper).

|  |  | Body Fat (%) | | | Skeletal Muscle (%) | | |
| --- | --- | --- | --- | --- | --- | --- | --- |
|  | Validity Measures | DXA | Samsung | InBody | DXA | Samsung | InBody |
| <60kg | Count | 28 | 28 | 28 | 28 | 28 | 28 |
|  | Mean | 22.81 | 24.34 | 18.04 | 32.65 | 39.6 | 45.09 |
|  | Standard Deviation | 7.86 | 3.69 | 4.71 | 2.91 | 2.86 | 3 |
|  | MAE |  | 2.7 | 4.84 |  | 6.95 | 12.44 |
|  | MAPE |  | 12.93 | 21.54 |  | 21.72 | 38.61 |
|  | Correlation (*r*) |  | 0.74 | 0.84 |  | 0.65 | 0.66 |
|  | Correlation (CCC) |  | 0.67 | 0.55 |  | 0.16 | 0.06 |
|  | Deming Regression Intercept |  | 8.57 | -3.93 |  | 7.81 | 11 |
|  | Deming Regression Slope |  | 0.69 | 0.96 |  | 0.97 | 1.04 |
|  | Bland-Altman Mean Bias (95% CI) |  | -1.53 (-2.80, -0.26) | 4.77 (3.72, 5.82) |  | -6.95 (-7.89, -6.02) | -12.44 (-13.39, -11.50) |
|  | Bland-Altman Lower LOA |  | -7.95 | -0.53 |  | -11.68 | -17.22 |
|  | Bland-Altman Upper LOA |  | 4.89 | 10.07 |  | -2.23 | -7.66 |
| 60-70kg | Count | 32 | 32 | 32 | 31 | 31 | 31 |
|  | Mean | 23.87 | 25.16 | 19.4 | 33.1 | 39.87 | 45.32 |
|  | Standard Deviation | 9.04 | 7.59 | 8.98 | 4 | 4.68 | 5.71 |
|  | MAE |  | 2.98 | 4.49 |  | 6.78 | 12.22 |
|  | MAPE |  | 17.27 | 21.38 |  | 20.63 | 36.9 |
|  | Correlation (*r*) |  | 0.92 | 0.97 |  | 0.94 | 0.97 |
|  | Correlation (CCC) |  | 0.9 | 0.86 |  | 0.41 | 0.22 |
|  | Deming Regression Intercept |  | 5.43 | -4.3 |  | 0.77 | -2.47 |
|  | Deming Regression Slope |  | 0.83 | 0.99 |  | 1.18 | 1.44 |
|  | Bland-Altman Mean Bias (95% CI) |  | -1.29 (-2.59, 0.00) | 4.47 (3.65, 5.28) |  | -6.78 (-7.37, -6.18) | -12.22 (-12.99, -11.45) |
|  | Bland-Altman Lower LOA |  | -8.35 | 0.04 |  | -9.97 | -16.33 |
|  | Bland-Altman Upper LOA |  | 5.76 | 8.89 |  | -3.58 | -8.11 |
| 70-80kg | Count | 21 | 21 | 21 | 21 | 21 | 21 |
|  | Mean | 24.58 | 25.3 | 19.65 | 33.3 | 40.02 | 45.32 |
|  | Standard Deviation | 10.24 | 8.96 | 10.41 | 5.31 | 5.54 | 6.36 |
|  | MAE |  | 2.72 | 4.95 |  | 6.72 | 12.01 |
|  | MAPE |  | 13.79 | 23.54 |  | 20.84 | 36.63 |
|  | Correlation (*r*) |  | 0.94 | 0.97 |  | 0.91 | 0.96 |
|  | Correlation (CCC) |  | 0.92 | 0.87 |  | 0.51 | 0.3 |
|  | Deming Regression Intercept |  | 3.98 | -5.35 |  | 5.08 | 5.09 |
|  | Deming Regression Slope |  | 0.87 | 1.02 |  | 1.05 | 1.21 |
|  | Bland-Altman Mean Bias (95% CI) |  | -0.72 (-2.39, 0.95) | 4.93 (3.83, 6.04) |  | -6.72 (-7.76, -5.68) | -12.01 (-12.88, -11.15) |
|  | Bland-Altman Lower LOA |  | -7.92 | 0.18 |  | -11.19 | -15.74 |
|  | Bland-Altman Upper LOA |  | 6.48 | 9.69 |  | -2.24 | -8.28 |
| 80-90kg | Count | 13 | 13 | 13 | 13 | 12 | 13 |
|  | Mean | 25.45 | 25.32 | 19.78 | 34.04 | 39.86 | 45.76 |
|  | Standard Deviation | 8.8 | 7.62 | 8.72 | 4.06 | 4.37 | 5.29 |
|  | MAE |  | 2.43 | 5.66 |  | 6.01 | 11.72 |
|  | MAPE |  | 12.72 | 24.98 |  | 18.03 | 34.61 |
|  | Correlation (*r*) |  | 0.94 | 0.98 |  | 0.92 | 0.95 |
|  | Correlation (CCC) |  | 0.93 | 0.8 |  | 0.45 | 0.21 |
|  | Deming Regression Intercept |  | 3.48 | -5.43 |  | 4.43 | 0.9 |
|  | Deming Regression Slope |  | 0.86 | 0.99 |  | 1.05 | 1.32 |
|  | Bland-Altman Lower LOA |  | -5.81 | 2.34 |  | -9.29 | -15.39 |
|  | Bland-Altman Upper LOA |  | 6.06 | 8.98 |  | -2.72 | -8.05 |
| >90kg | Count | 14 | 14 | 14 | 14 | 14 | 14 |
|  | Mean | 35.46 | 35.23 | 32.53 | 29.56 | 34.41 | 38.16 |
|  | Standard Deviation | 10.89 | 8.63 | 11.5 | 6.21 | 5.13 | 7.08 |
|  | MAE |  | 3.59 | 3.86 |  | 4.85 | 8.6 |
|  | MAPE |  | 12.95 | 14.14 |  | 18.11 | 30.15 |
|  | Correlation (*r*) |  | 0.93 | 0.95 |  | 0.94 | 0.94 |
|  | Correlation (CCC) |  | 0.91 | 0.92 |  | 0.67 | 0.49 |
|  | Deming Regression Intercept |  | 7.6 | -4.99 |  | 10.3 | 4.21 |
|  | Deming Regression Slope |  | 0.78 | 1.06 |  | 0.82 | 1.15 |
|  | Bland-Altman Mean Bias (95% CI) |  | 0.23 (-2.23, 2.69) | 2.93 (0.92, 4.94) |  | -4.85 (-6.12, -3.58) | -8.60 (-10.01, -7.19) |
|  | Bland-Altman Lower LOA |  | -8.12 | -3.91 |  | -9.16 | -13.4 |
|  | Bland-Altman Upper LOA |  | 8.58 | 9.76 |  | -0.54 | -3.8 |
